# Rituximab induced cytokine release syndrome in an MS patient: A case report

**DOI:** 10.1002/ccr3.4407

**Published:** 2021-07-09

**Authors:** Masoud Etemadifar, Mehri Salari, Mahdieh Saeri, Amirhossein Akhavan Sigari, Sara Ebrahimi Pelarti

**Affiliations:** ^1^ Department of Neurosurgery Alzahra University Hospital Isfahan University of Medical Sciences Isfahan Iran; ^2^ Functional Neurosurgery Research Center Shohada Tajrish Comprehensive Neurosurgical Center of Excellence Shahid Beheshti University of Medical Sciences Tehran Iran; ^3^ Alzahra Research Institute Alzahra University Hospital Isfahan University of Medical Sciences Isfahan Iran

**Keywords:** cytokine release syndrome, multiple sclerosis, rituximab

## Abstract

Cytokine release syndrome with rituximab has been reported in certain diseases, however, it is rarely reported in MS patients treated with rituximab. The treating physician should suspect the syndrome when typical signs and symptoms appear.

## Graphical abstract

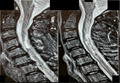

## 1 BACKGROUND

Rituximab use in multiple sclerosis has been promising. Cytokine release syndrome (CRS) is a common side effect of rituximab in patients with lymphoma. We report a case of a 44‐year‐old man with a history of relapsing‐remitting multiple sclerosis, who presented with signs and symptoms consistent with CRS after rituximab initiation.

Multiple sclerosis (MS) is an immune‐mediated inflammatory disease of the central nervous system (CNS). Lymphocytes play a key role in the pathogenesis of MS. Due to the critical role of B lymphocytes in MS pathology, monoclonal antibodies targeting such cells have been proposed for MS treatment.^1^ Rituximab, a monoclonal antibody against CD20 receptors on B lymphocytes, is now being widely used for autoimmune diseases such as MS, rheumatoid arthritis, and cancer therapy and has had comparable clinical outcomes in managing MS than other injectable and oral disease‐modifying therapies (DMTs). ^2^ Therefore, rituximab is being prescribed by medical practitioners for MS therapy in certain countries.^1^

Infections and mild to moderate infusion‐related reactions are more common side effects of rituximab and life‐threatening complications such as anaphylaxis appear to be less frequent. An important side effect of rituximab is cytokine release syndrome (CRS).^3^, ^4^ CRS is a systemic inflammatory response that occurs following an extensive release of inflammatory cytokines secondary to the activation of myeloid cells and lymphocytes and is mainly characterized by rash, fever, mental status changes, etc which is more apparent in patients undergoing immunotherapy for malignancies especially lymphomas.^5^ CRS has been reported in MS patients treated with monoclonal antibodies such as alemtuzumab, however, rituximab‐induced CRS in MS patients is extremely rare.^6^

## 2 CASE PRESENTATION

A 44‐year‐old man with a history of relapsing‐remitting multiple sclerosis was referred to our MS Clinic for the evaluation of bilateral edema of the lower limbs. The edema appeared shortly after the patient had received his last rituximab infusion. The patient also complained of headache and generalized arthralgia. Upon physical examination bilateral symmetric pitting edema of the lower limbs was evident, no skin lesions were present, vital signs were stable and the patient was not febrile. Lab data revealed an increased serum creatinine level of 1.4 mg/dL, SGPT of 78 U/l (N = 6‐45), SGOT of 64 U/l (N = 8‐40), along with a C‐reactive protein (CRP) level of 57 mg/dL (N = less than 10 mg/dL). The patient had normal hemoglobin, hematocrit, white blood cell count, and mildly decreased platelet counts (13.6 mg/dl, 44%, 5600 × 10^9^/L, and 123000/μL, respectively).

**FIGURE 1:**
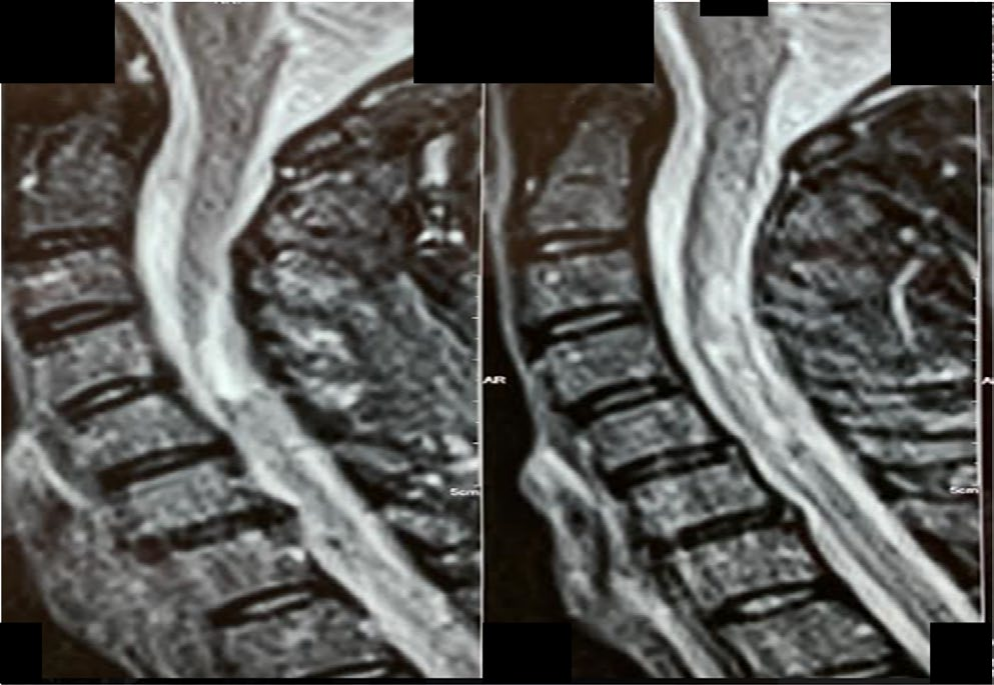
Fluid‐attenuated inversion recovery (FLAIR) cervical spine MRI of the patient revealing a hyperintense lesion at the level of C3‐C4

On 2001, the patient had experienced a right side optic neuritis, one year later he had ataxia and diplopia and magnetic resonance imaging (MRI) of the brain and spinal cord were done, which showed hyperintense lesions at the level of the cervical spinal cord (Figure 1), the patient was diagnosed with MS and interferon‐beta 1a was started. Five years later, he had an attack of bilateral lower limb paresis, and 3 years later, he experienced a severe attack of quadriparesis. After the acute management of his attack, the patient's drug was changed to fingolimod. On 2018, when he was on fingolimod for 2 years his drug was changed to rituximab by another neurologist. After the first dose of rituximab (Zytux™) (1 gr intravenous (IV) infusion (inf)), he experienced a severe infusion reaction, presented by erythema and urticarial lesions. Two days later, he noticed bilateral edema of the lower limbs that had gradually worsened and somnolence. The patient was evaluated by several specialists regarding his limb edema. His symptoms improved with antihistamines and corticosteroids, but after 6 months, when he receives his second dose of rituximab (1 gr IV inf.), he presented again with bilateral limb edema and the symptoms described above. Consult with an infectious disease specialist was done to rule out any infectious causes or sepsis.

Based on the patient's history and by taking into account lab data, cytokine release syndrome was highly suspected. The patient was managed with antihistamines and corticosteroids. Oral prednisolone of about 75 mg/day (1.5 mg/kg) was started for the patient and titrated over the course of four weeks. The patients symptoms were improved in two weeks; however, limb edema was completely resolved on week four of follow‐up.

## 3 DISCUSSION AND CONCLUSION

One of the main side effects of rituximab are infusion reactions, which mainly consist of cytokine release syndrome and in some cases, type I hypersensitivity reaction.^1^ Cytokine release syndrome is an overreaction of T lymphocytes that leads to an increased release of certain cytokines (interleukin (IL)‐1, IL‐2, IL‐6, IL‐8, IL‐10, tumor necrosis factor (TNF), and interferons (INF)) especially IL‐6. Lab work may reveal azotemia, hyperbilirubinemia, and elevated D‐dimer levels. CRS symptoms mainly consist of rash, fever, myalgia, arthralgia, nausea, vomiting, diarrhea, tachycardia, tachypnea, headache, confusion, mental change, and seizure.^7^, ^8^ Infection with COVID‐19 also induces CRS by massive release of cytokines sometimes stated as a “cytokine storm“.^9^ Tocilizumab is considered one of the drugs for treating CRS.^10^

In our case, middle‐aged man who was diagnosed with MS was started on rituximab. After the first dose the patient had shown signs of CRS; however, due to its rare prevalence, management was done based on a diagnosis of moderate allergic reaction. After the second dose of rituximab, the patients had presented with similar symptoms, although more severe. In another case report, a 72‐year‐old man was started on rituximab for diffuse large B‐cell lymphoma, and 18 hours after infusion the patient developed severe abdominal pain, refractory hypotension, and lactic acidosis. The patient eventually died and the authors attribute the symptoms and the cause of death to cytokine release syndrome induced by rituximab.^11^ Another case report, describes a 10‐year‐old boy with postbone marrow transplant Epstein‐Barr virus infection. The patient was started, therefore, on rituximab and developed refractory hypotension and a spiking fever within an hour of infusion. The patient did not have a lymphoproliferative disorder similar to our case.^12^ Tournamille et al also reports a case of a 46‐year‐old patient with familial cardiomyopathy who died due to CRS a few minutes after the administration of his second dose of rituximab.^13^ However, to the best of our knowledge, no reports of CRS induced by rituximab in an MS patient are currently available.

Neurologic side effects such as mental status changes can appear during or after other signs and symptoms.^14^ CRS has been classically associated with therapeutic monoclonal antibody infusions, most notably antiCD3 (OKT3), antiCD52 (alemtuzumab), and antiCD20 (rituximab)..^15^

An important differential diagnosis of CRS is capillary leak syndrome, serum sickness (type III hypersensitivity reaction),^16^ and idiopathic infusion reaction. Capillary leak syndrome is a massive leakage of fluid into the interstitial space secondary to increased permeability of vessels mediated by released interleukins that leads to sudden hypotension and shock. Other features may include generalized edema, hemoconcentration, and hypoalbuminemia. It is mainly seen in patients with sepsis.^4^ There are also reports of capillary leak syndrome in NMO patients treated with rituximab.^17^ A relatively new entity called the Kaposi sarcoma‐associated herpesvirus (KHSV) inflammatory cytokine syndrome (KICS) has also been described in recent paper. The syndrome occurs in patients with Human Immunodeficiency Virus (HIV) and concurrent Kaposi sarcoma and is characterized by pancytopenia, lymphadenopathy, and systemic inflammation.^18^

The overlap between CLS and CRS mandates differentiation of these two syndromes when approaching patients. Capillary fluid leakage is seen in both syndromes but the leakage in CLS is so severe that causes hypotension and shock. Cytokine release syndrome mostly starts with fever, rash, and malaise and is rarely associated with shock. However, hypotension can occur in the course of the disease leading to capillary leak syndrome.^8^

Management of CRS can be somewhat challenging. Although severe CRS can be lethal, other forms can mainly be managed with corticosteroids and general supportive care (fluid resuscitation, antipyretics for fever, supplemental oxygen, etc). Tocilizumab which is a monoclonal antibody against IL‐6 receptor is also used for the management of CRS where IL‐6 plays a central role in its pathology. Tocilizumab is preferred over corticosteroids in some cases, however, as steroids can potentially blunt the original treatment for the primary disease.^19^ Anticytokine therapy and prophylactic dexamethasone can also be started as a preventative task after immunotherapy initiation when cytokine levels are presumed to elevate based on serial measurements, mainly in cases being treated for lymphoma where likelihood of CRS is high. Remission of CRS usually occurs up to three days therapy.^20^

Although rituximab‐induced cytokine release syndrome has been reported in the treatment of certain diseases, this syndrome is very rare in MS patients being treated with the drug. Monoclonal antibodies such as rituximab are now being widely used to treat MS and all side effects of the drug should be assessed and differentials should be recognized when adverse effects occur. Therefore, cautious use of the drug in this subset of patients is advised.

## CONFLICT OF INTEREST

The authors have no competing interests to declare.

## AUTHOR CONTRIBUTIONS

ME, MeS, and MaS provided the case's relevant information, history, and images. AAS and SE wrote the primary draft of the manuscript. AAS, MeS, ME, and MaS wrote the final draft and extracted relevant data from the literature. The final manuscript was read and approved by all of the authors.

## ETHICAL APPROVAL

Written consent was obtained from the patient for the presentation of this case.

## CONSENT FOR PUBLICATION

Written consent was taken from the patient for the publication of this paper. A copy of the consent form is available for review by the editor of the journal.

## Data Availability

Not applicable.
